# Internet-delivered cognitive behavioral therapy for adolescents with insomnia: Feasibility and preliminary efficacy

**DOI:** 10.1177/13591045231202426

**Published:** 2023-09-12

**Authors:** Li Åslund, Susanna Jernelöv, Eva Serlachius, Sarah Vigerland, Rikard K Wicksell, Eva Henje, Mats Lekander

**Affiliations:** 1 Department of Clinical Neuroscience, 27106 Karolinska Institutet, Sweden; 2 Centre for Psychiatry Research, Department of Clinical Neuroscience, 27106 Karolinska Institutet, Sweden; 3 Child and Adolescent Psychiatry, Department of Clinical Sciences, Faculty of Medicine, 5193 Lund University, Sweden; 4 Child and Adolescent Psychiatry, Region Skåne, Sweden; 5 Pain Clinic, Capio St Göran Hospital, Sweden; 6 Department of Clinical Science, 59588 Umeå University, Sweden; 7 Stress Research Institute, Stockholm University, Sweden

**Keywords:** Adolescent, digital intervention, cognitive-behavioral therapy, comorbidity, feasibility, insomnia disorder, psychiatric disorders

## Abstract

**Background:**

Insomnia is common in adolescents. This study evaluated feasibility and preliminary efficacy of a six-week internet-delivered cognitive-behavioral therapy for insomnia (ICBT-I) in adolescents.

**Methods:**

In this uncontrolled pilot study, participants (*n* = 27, 78% female) completed assessments pre- and post intervention. Data on recruitment, adherence to treatment, treatment activity, satisfaction and credibility was collected to assess feasibility. Self-reported insomnia symptoms, sleep parameters as well as depression, anxiety and daytime function were also assessed.

**Results:**

Participants showed good adherence to treatment and found the intervention overall credible and satisfactory. From pre- to post-assessment, statistically significant improvements were found for insomnia symptoms (*p* < .001; d = 1.02), sleep onset latency (*p* < .001; d = .39), wake after sleep onset (*p* = .001; d = .34), sleep efficiency (*p* < .001; d = .5) and depression (*p* = .01, d = .37). Changes in scores of total sleep time, generalized anxiety, daytime sleepiness and functional disability were not significant.

**Conclusions:**

The present study indicates that ICBT-I is well accepted by adolescents, that insomnia symptoms and sleep parameters can improve following the intervention, and that co-morbid symptoms of depression can be reduced. Due to the limited sample size and the uncontrolled design, the suggested results need to be replicated in well-powered controlled clinical trials.

## Introduction

Sleep disorders, notably insomnia, are common in adolescence. Insomnia is defined as difficulties falling asleep, staying asleep, and/or suffering from early awakenings for at least three nights/week over at least three months with associated clinically significant functional daytime impairment (American Psychiatric Association, 2013). Insomnia often debuts with prolonged sleep onset at a median age of 11 (Johnson et al., 2006). About 10% of adolescents meet criteria for an insomnia diagnosis (Dohnt et al., 2012) and more than 30% report “at least some” symptoms of the disorder (Dohnt et al., 2012).

There is a co-occurrence of insomnia and depressive symptoms in adolescence, where 66% of adolescents with insomnia have been shown to present symptoms of depression (Amaral et al., 2017). This co-morbid symptomatology is also true for insomnia and anxiety disorders where the risk of developing disrupted sleep and anxiety increases as children go from late childhood into adolescence (McMakin & Alfano, 2015). When examining children and adolescents between 7–15 years with major depressive disorders both female (77%) and male (69%) reported concurrent symptoms of sleep disturbances (Liu et al., 2007). In children with anxiety disorders, 90% reported concurrent sleep disturbances (Chase & Pincus, 2011). Among adolescents with comorbid disorders, anxiety disorders preceded insomnia in 73% of the cases, while insomnia preceded major depression disorder in 69% of the comorbid cases (Johnson et al., 2006).

Cognitive behavioral therapy (CBT) for insomnia (CBT-I) is indicated to show positive effects on insomnia and several sleep parameters in adolescent populations (Aslund et al., 2018; Meltzer & Mindell, 2014). CBT-I refers to a number of techniques, both cognitive and behaviorally oriented, that aim to improve the participant’s sleep (Edinger & Means, 2005). This includes sleep restriction, stimulus control, and changing negative thoughts about sleep through cognitive interventions and relaxation (Epstein et al., 2012; Morin et al., 1999). The intervention has been shown effective in several modes of delivery, for example, individual face-to-face treatment, group treatment and self-help support via books (Gao et al., 2022; Jernelov et al., 2012). CBT-I can also be delivered via an internet platform, and can then be denoted internet-delivered cognitive behavioral therapy for insomnia, ICBT-I (Ye et al., 2015). ICBT-I is founded on the same principles and techniques as face-to-face CBT-I, but the intervention is offered online, often with therapist support. In adults, ICBT-I improves sleep quality and has a positive effect on insomnia severity, daytime sleepiness and co-morbid depression and anxiety (Kaldo et al., 2015; Strom et al., 2004; Vincent & Lewycky, 2009; Ye et al., 2015). In adolescents, studies are scarce but there are indications of a positive effect of internet-delivered CBT-I on insomnia and sleep efficiency, sleep onset latency, wake after sleep onset and total sleep time (de Bruin et al., 2015). Since adolescents are more reluctant than adults to seek help for their psychological problems (Cheng, 2009), easy access to treatment over the internet can be an important factor for this age-group. Assessment of the effect of internet-delivered CBT for various psychiatric and somatic conditions in children and adolescents has demonstrated that the adaptation to internet-delivered format is feasible and acceptable in this age group (Vigerland et al., 2016).

Based on this, internet-delivered CBT-I, is a compelling treatment option for this age group. However, before conducting a large, randomized controlled trial or implementing a treatment in a health care setting, evaluation of issues related to feasibility and acceptability must be addressed. These include difficulties with participant recruitment in the setting of health care, participants not actively taking part in the intervention and/or failing to adhere to treatment over time and participants not finding that the treatment offered is relevant or correspond to their needs.

This study therefore aimed to explore the participant feasibility of ICBT-I, with regards to recruitment, adolescents’ adherence to treatment, treatment activity, satisfaction and credibility. Another aim was to test the preliminary efficacy of ICBT-I on insomnia symptom severity, sleep onset latency (SOL), wake after sleep onset (WASO), total sleep time (TST), sleep efficiency (SE), and symptoms of depression, anxiety and daytime function (disability and sleepiness).

## Methods

### Design and ethics

The study was an uncontrolled clinical pilot study where all included participants underwent treatment. At pre-treatment, participants were assessed on treatment feasibility (credibility), insomnia symptoms, sleep, comorbid psychiatric symptoms and daytime functioning. At post-treatment, additional measures of treatment feasibility (adherence to treatment, participant and therapist activity, treatment satisfaction) were assessed, together with the same measures of insomnia symptoms, sleep, comorbid symptoms and daytime functioning as pre-treatment. The Regional Ethical Review board of Stockholm approved the study (2017/2315-31/2). Participants and guardians gave written consent per Helsinki Declaration.

### Participants

Participants were aged between 13–17 years with adequate Swedish language skills. All participants met criteria for insomnia disorder according to DSM-V (American Psychiatric Association, 2013). Exclusion criteria were severe psychiatric disorders (e.g. bipolar disorder, psychotic disorder, autism spectrum disorder), severe suicidality (defined as ≥17 points on MINI-KID suicide subscale), substance use disorder, any on-going use of psychotropic medication and any ongoing psychological treatment for insomnia or other psychiatric disorder.

### Recruitment procedure and data collection

**Figure 1.**
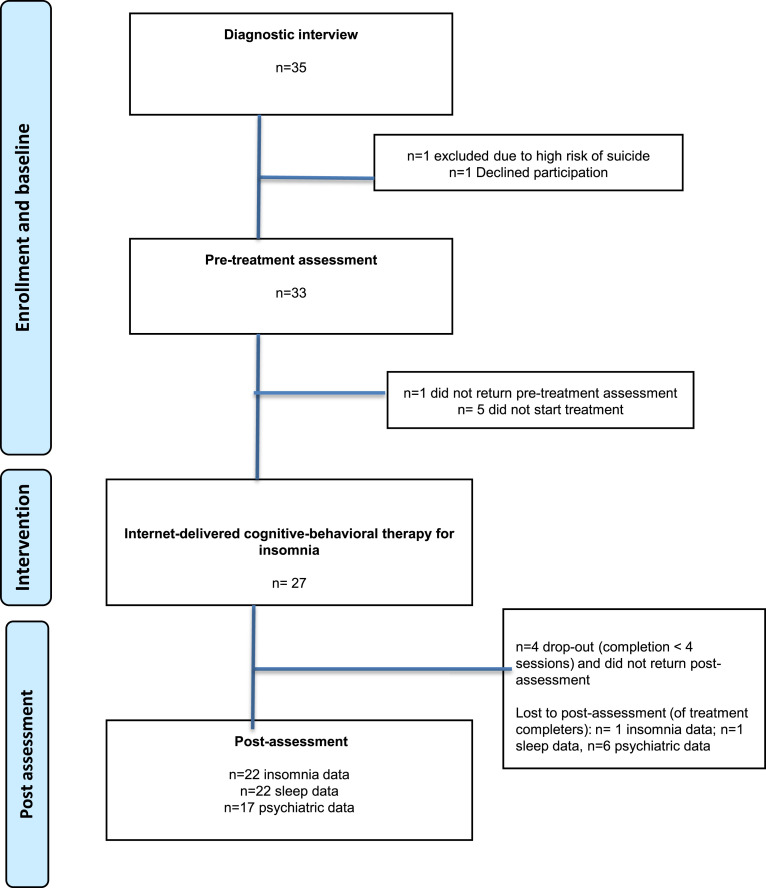
Flow chart.

Participants (*n* = 27) were recruited in Stockholm between September 2018 and November 2019 through various channels. Initially, recruitment hinged on clinical and school health care referrals, later broadened to include newspaper ads. For details, see “Measures”. Prospective participants expressed their interest by submitting their guardian’s contact information via an online registration form, subsequently receiving a prompt call from a clinical psychologist for a brief telephone screening. The screening involved an evaluation of factors including insomnia symptoms, current psychological and/or pharmacological treatments, and psychiatric disorders that met the criteria for exclusion. If considered eligible, participants were invited to an interview together with their parents/guardians. Following the interview, individuals who met criteria for inclusion were offered study participation. Participants completed online baseline assessments (sleep-wake diary and questionnaires on insomnia, sleepiness, psychiatric symptoms and function), see Figure 1. No compensation was offered to the participants.

### Intervention: iSNOOZE

The CBT-I intervention used in the present study, called iSNOOZE, was based on ySNOOZE, a face-to-face cognitive-behavioral intervention for youth insomnia [9]. iSNOOZE spanned over 6 weeks and included six digital modules directed at the adolescent participant, who logged in on a secure platform in order to access treatment. Parents/caregivers were informed about the treatment through their own platform login but did not take active part in the intervention and could not access the communication between the participant and the therapist. The modules consisted of text, illustrations and audio files with embedded homework exercises. Participants were encouraged to log in at least once a week to complete the module corresponding to that week, something that would require around 30 minutes in total of reading, listening, and writing. The main component of the treatment was sleep restriction, introduced in module 2 and adjusted weekly based on calculated SE from digital sleep-wake diary registrations kept by participants throughout the treatment. If SE, that is proportion of time in bed spent asleep, was 85% or higher, time in bed was extended by 15 minutes, and if SE was 80% or lower, time in bed was curtailed by 15 minutes. Adjustments were done primarily by changing bedtimes, whereas rise-times were preferably kept constant. The minimal sleep window was set to 5 hours. A more detailed description of the iSNOOZE content is presented in Supplementary Table 1. Participants presenting suicidal ideations, or other clinical issues, were handled according to the regular routines of the research clinic, as part of study procedure. Participants received therapist support three times per week through messages on the platform, including guidance, feedback on homework assignments and answers to questions from the participants. Text messages were used to remind participants to log on to the platform but not to provide therapy. Therapists (*n* = 2) were clinical psychologists with >18 months of CBT-training and a half-day workshop on using iSNOOZE. Clinical supervision was provided by the first author throughout the study.

### Measures

The insomnia diagnosis was established using a structured interview based on DSM-V diagnostic criteria for sleep disorders (American Psychiatric Association, 2013). The Mini International Neuropsychiatric Interview for Children and Adolescents, MINI-KID (Sheehan et al., 1998), a semi-structured diagnostic interview based on DSM-IV, was used to diagnose comorbid psychiatric disorders.

#### Primary outcome: Treatment feasibility

**Recruitment** was assessed by monitoring both the *quantity and rate* of participant recruitment across various channels (primary care, secondary care, school health care and newspaper advertisement).

**Adherence to treatment** was assessed post-treatment though *completion rate* (i.e., how many modules of the treatment each participant finished) together with the *ratio of treatment completers* (defined as participants that finished ≥4 out of the 6 modules).

**Participant and therapist activity in treatment** was assessed post-treatment by *participants’ activ*ity on the platform (i.e., number and frequency of log-ins, time spent on platform) and *therapists’ activity* (time spent on each participants).

**Treatment satisfaction** was measured post-treatment by the *Client Satisfaction Questionnaire – child version (CSQ-C)* (Attkisson & Zwick, 1982), a 8-item self- and parent rated scale measuring different aspects of satisfaction with treatment. Participants rate their experience on a 4-grade scale, generating a total score between 8 and 32 (higher score indicating higher satisfaction). For each question, rating options depend on the dimension, for example, for “Quality of the treatment” participants can choose between “poor”, “mediocre”, “good” or “excellent” and for “Satisfaction with treatment” the choice stands between “completely unsatisfied”, “somewhat unsatisfied”, “mostly satisfied” and “very satisfied”.

**Treatment credibility** was measured mid- and post-treatment by the *Treatment credibility and expectancy – Child version (C-scale C)* (Borkovec TD), a 5-item scale measuring the degree (from 1–10, with higher number indicating higher credibility/expectancy, total score 50) to which participants rate their expectancies regarding the logic, credibility and possible symptom improvement following an intervention.

#### Secondary outcomes: Insomnia, sleep, comorbid psychiatric symptoms and daytime functioning

**Insomnia symptom severity** was measured pre- and post-treatment by *the Insomnia Severity Index-adolescent version* (ISI–a) (Kanstrup et al., 2014), a seven items self-report questionnaire with scores ranging from 0 to 28, with higher scores indicating more severe insomnia. The adult version of the Insomnia Severity Index (ISI) is a well-used scale with adequate psychometric properties (Chronbach’s α = .74) and sensitivity to change (Bastien et al., 2001; Blais et al., 1997). Clinical cut-off for improvement after treatment of adult insomnia is −9.9 points (marked) and −8.4 (moderate) respectively, and a score below 8 indicates remission (Morin et al., 2011). ISI-a is an adapted version for adolescents with similar psychometric properties (Chronbach’s α = .88; (Kanstrup et al., 2014).

**Subjective sleep** (SOL, WASO, TST and SE) was measured pre- and post-treatment through a self-report sleep wake diary (SWD; Carney et al., 2012; Short et al., 2017) during 7 consecutive nights.

**Psychiatric symptoms**: Anxiety and depression symptoms were measured pre- and post-treatment by *Revised Children’s Anxiety and Depression Scale – Child and Parent version* (RCADS-C/P; (Chorpita et al., 2000). RCADS_C/P is a child and parent self-report measure of anxiety- and depression related psychopathology. The 48 items of the RCADS-C/P measures five domains of anxiety: generalized anxiety, panic anxiety, separation anxiety, social anxiety and obsessive-compulsive anxiety, as well as symptoms of depression. The four-graded scale ranges from 0 = “Never” to 3 = “Always”. The RCADS C/P has been shown to hold strong psychometric properties, with high internal consistency for the total score as well as the subscales, satisfactory test-retest reliability and good criterion validity (Chorpita et al., 2005; Ebesutani et al., 2010).

**Daytime functioning** was measured pre- and post-treatment with *the Functional Disability Inventory* (FDI; Walker & Greene, 1991), a 15 item self-report questionnaire with scores ranging from 0 (no disability) to 60 (severe disability) and the *Pediatric Daytime Sleepiness Scale (PDSS;* (Drake et al., 2003), a 8 item self-rated questionnaire to measure daytime sleepiness, with scores ranging from 0 to 4 (higher score equals higher levels of sleepiness).

### Statistical analyses

Multiple imputations using SPSS 25 were performed for missing data at post-treatment assessments, with missing value analyses and assessment of data to ensure normal distribution. Paired samples t-tests were performed (intent-to-treat) to assess whether changes in scores from pre- to post-treatment were statistically significant (*p* > .05). The effect sizes of within-group changes were calculated as standardized Cohen’s *d* effect sizes (Cohen, 1992) with 95% confidence intervals. The within-group effect was considered small if *d* < .5, moderate if > .8, and large if > 1.1 (Öst LG, 2006).

## Results

### Sample and study attrition

For demographic information and baseline data for the 27 participants, see Table 1. A majority of participants (78%) were female. The majority of participants (*n* = 17) did not report any comorbid psychiatric disorder according to MINI-kid. For those who reported comorbidity, the average number of diagnoses was 1.6, with the most common being generalized anxiety disorder and specific phobia. At post-treatment, assessments were completed by 81% (insomnia and measures of sleep) and 63% (psychiatric symptoms) of the original sample. Participants who dropped out of treatment (*n* = 4) did not return post-data.

**Table 1.** Demographic data for *n* = 27 participants.

Age, mean (SD) years	15.2 (1,7)
Female, *n* (%)	21 (78)
Male, *n* (%)	6 (22)
Major depressive disorder, *n* (%)	1 (4)
Generalized anxiety disorder, *n* (%)	4 (15)
Social phobia, *n* (%)	2 (7)
Panic disorder, *n* (%)	2 (7)
Agoraphobia disorder, *n* (%)	1 (4)
Specific phobia, *n* (%)	4 (15)
ADHD, *n* (%)	2 (7)

Note. ADHD: Attention Deficit Hyperactivity Disorder.

### Treatment feasibility

#### Recruitment

Participants (*n* = 27) were recruited during a total of 14 months. Most participants (*n* = 23) were recruited through advertisement featured in a local newspaper seven months into the trial and only a few through secondary care clinics (*n* = 4). No participants were recruited through primary care clinics or school health care.

#### Adherence to treatment

23 of the 27 participants (85%) completed ≥4 out of the 6 modules of the program and were thus considered treatment completers. The mean number of completed modules was 5.22 (all participants) and 5.61 (completers). All participants completed at least half of the modules and 16 participants (59%) completed all six modules.

#### Participant and therapist activity

Completers logged in on average 5.32 times/week (range 2.2–10.17) and spent 2 hours and 33 minutes (range 24 min-10 h 48 min) per week logged on to the platform. Average time between login was 2.13 days (range 1–7 days). Therapist spent on average 19 minutes per week per participant.

#### Treatment satisfaction

Participants reported an overall mean score of 24.21 post-treatment. Regarding participants ratings of “good” and “excellent” on individual questions, the span was between 47% of participants choosing one of these options (for the extent that the program met the user’s needs) to 89% of participants (for recommendation of program to a friend, quality of the service received and amount of help received). 79% of the adolescents reported that they were “satisfied” or “very satisfied” with the overall intervention.

#### Treatment credibility

Participants reported good overall (mean 36.96) treatment credibility pre-treatment, with sub-scores ranging from 6.86 to 8.6. Post-treatment, scores ranged from 7.55 to 8.26. The numerically largest change in score from pre- to post assessment was +1.04 for the subscale “recommend treatment to a friend”.

### Preliminary treatment efficacy

#### Changes in insomnia symptom severity and sleep pre-to post-treatment

**Figure 2.**
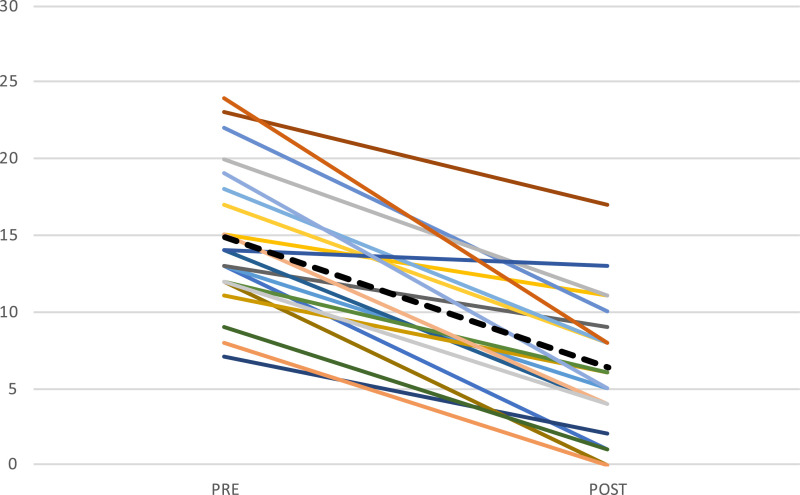
ISI-a score for each participant who presented data at pre and post assessment (*n* = 22). Note. Mean score presented as dotted line.

Means, standard deviations, and effect size with 95% CI for all outcomes are reported in Table 2. There was a statistically significant improvement in insomnia symptoms from pre- to post-treatment (t (61) = −8.39, *p* < .001, *d* = 1.02) as measured by the ISI-a. For details on ISI-a scores of each participant, see Figure 2.

**Table 2.** Outcome measures pre- and post treatment.

Measure	Pre-treatment (*n* = 27)		Post-treatment (*n* = 27)		Pre-to post-treatment					Effect size Cohen’s d
	Mean	SD	Mean	SD	Mean change	*St. Mean Err*	95% conf. Interval		*p*-value	
							Cl-	CI+		
ISI-a	14.95	4.69	6.56	4.41	−8.39	.81	6.67	9.91	<.001	1.02
SOL SWD	72.91	44.96	36.50	23.29	−36.41	8.89	17.32	52.20	<.001	.39
WASO SWD	25.90	31.93	6.23	8.30	−19.67	5.69	8.00	30.31	.001	.34
TST SWD	402.98	66.22	423.26	86.89	20.28	21.39	−68.41	16.95	.223	.12
SE SWD	.77	.12	.86	.08	.09	.02	−.15	−.06	<.001	0.5
PDSS	6.06	1.60	5.41	1.46	−.65	.60	−.83	1.92	.39	.09
RCADS-C total	44.96	21.11	41.76	28.60	−3.20	2.69	−3.46	7.55	.45	.08
RCADS-C depression	11.89	5.23	9.33	5.83	−2.56	.70	1.15	3.96	.001	.37
RCADS-C GAD	6.15	4.29	5.68	4.75	−.47	.43	−.37	1.31	.28	0.1
FDI	1.29	1.64	1.49	1.45	.20	.35	−.90	.51	.58	.05
CSQ-C**	n/a	n/a	24.21	4.13	n/a	n/a	n/a	n/a	n/a	n/a
C-scale-C	36.96	6.67	n/a	n/a	n/a	n/a	n/a	n/a	n/a	n/a

Note. Means, measures of variance, 95% CI’s, and within group effect sizes for pre- and post-assessments with missing values replaced by multiple imputation.

ISI-a: Insomnia Severity Index-adolescent version; SOL: sleep onset latency; WASO: wake after sleep onset; TST: total sleep time; SE: sleep efficiency; SWD: Sleep Wake Diary; PDSS: Pediatric Daytime Sleepiness Scale; RCADS-C/P: Revised Children’s Anxiety and Depression Scale- Child/Parent; FDI: Functional Disability Inventory; CSQ-C: Client Satisfaction Questionnaire-Child; **n = 19; n/a: not applicable.

There was also a statistically significant improvement in sleep measures from pre- to post treatment for SOL (t (3.91) = -36.41, *p* < .001, *d* = .39), WASO (t (3.37) = −19.67, *p = .001, d* = .34) and SE (t (−4.31) = .09, *p* < .001, *d* = .5) but not for TST.

#### Changes in comorbid symptoms and daytime function pre to post-treatment

Symptoms of depression (RCADS-C Depression) showed statistically significant improvements from pre- to post-treatment (t (44) = −2.56, *p* = .001, *d* = .37). Changes in scores of generalized anxiety (RCADS-C GAD), daytime sleepiness (PDSS) and functional disability (FDI) were not statistically significant (*p*’s > .28).

## Discussion

This feasibility study examined recruitment, adherence to treatment, patient and therapist activity, treatment satisfaction, and treatment credibility in internet-delivered CBT-I in adolescents with insomnia. In addition, preliminary efficacy of the intervention on insomnia symptoms, sleep onset latency, wake after sleep onset, total sleep time, sleep efficiency as well as comorbid symptoms of anxiety, depression and daytime function were assessed. Participant recruitment data suggest that relying solely on clinical samples for a larger randomized controlled trial might be unfeasible due to lacking partnerships with recruitment sites. Enhanced collaborations with clinical institutions could improve credibility and access to potential participants. Effective collaborations are vital for future intervention implementation in clinical care. Newspaper ads surpassed clinical settings for recruitment; exploring platforms like Facebook and TikTok is warranted. Tailored incentives considering participants' motivations could boost enrollment. More strategies can optimize full-scale trial recruitment for higher participation and trial success.

Regarding adherence to treatment, with a majority of participants considered treatment completers, was in line with what has been noted in CBT-I on adolescents with comorbid psychiatric disorders (Aslund et al., 2020). Unfortunately, it is quite common that participants do not adhere to CBT-I (Riedel & Lichstein, 2001) and the sleep restriction component is often perceived as highly aversive (Kyle et al., 2011). In adult CBT-I, it seems as if more therapist support can be beneficial for participants with for example, attention-deficit disorder to improve retention (Jernelov et al., 2019). As internet-delivered CBT-I lacks the physical interaction with a therapist, some participants might lack a more direct personal support to pursue the long-term goals of sleep restriction (Kyle et al., 2014). Having an easy-access chat-function where participants and therapist can interact on an almost daily basis might be one possible way to enhance support for higher treatment adherence. In the present study, participants were in general active on the platform and logged in on a regular basis, supporting acceptability of the treatment. For each treatment week and participant, therapists spent approximately 1/3 of the time traditionally devoted to face-to-face CBT-I (60 min/session). Using ICBT-I in clinical care could thus significantly increase the number of patients treated by each therapist and improve cost-effectiveness. Participant-reported treatment satisfaction and credibility was in line with previous studies on adolescent ICBT-I (Wickberg et al., 2022), suggesting that the intervention was well-suited for the study population.

The decrease in insomnia symptoms between pre- and post-assessments support a preliminary treatment efficacy of the intervention. This is in line with previous findings in adolescents (de Bruin et al., 2015) and aligns well with treatment results from adults (van Straten et al., 2017). The reduction of SOL and WASO, as well as the increase in SE from pre- to post assessment, give preliminary support for using ICBT-I in adolescents with insomnia disorder, corroborating previous indications (Aslund et al., 2018). For comorbid psychiatric symptoms and daytime function, a statistically significant reduction in depressive symptoms was observed after the intervention, with a small effect size. This is in line with what has been noted regarding CBT-I and adolescents in child- and adolescent psychiatry (Aslund et al., 2020). No statistically significant change was however noted for anxiety or daytime function (disability and sleepiness).

Regarding clinical significance, mean insomnia symptom severity was reduced from clinical levels pre-treatment to sub-clinical levels post-treatment with mean score reduction considered a clinically significant improvement in adults (Morin et al., 2011). Examining improvements in insomnia symptoms for each participant show marked or moderate improvements (Morin et al., 2011) and insomnia remission (Morin et al., 2011) for the majority of the participants. For sleep data post-treatment, average SOL was still slightly higher than the relevant cut-off for insomnia disorder (Lichstein et al., 2003) but below what is regarded as an indicator of poor sleep (Ohayon et al., 2017). Average WASO and SE were no longer indicative of poor sleep (Lichstein et al., 2003; Ohayon et al., 2017). Mean TST increased by 20 minutes, but this change was not statistically significant, possibly due to low power. For co-morbid psychiatric symptomatology, it should be noted that average scores for all comorbid psychiatric symptoms pre-treatment were below cut-off for clinical significance (Chorpita et al., 2000). This was also true for daytime function (Kashikar-Zuck et al., 2011) and sleepiness (Meyer et al., 2017). It is possible that these low levels do not permit a substantial change in comorbid symptoms between pre- and post intervention and a floor effect is thus indicated. Overall, the examination of clinical significance with regard to change in symptoms following ICBT-I, as well as symptom-levels post-treatment, indicate that participants may benefit from the intervention both with regard to insomnia symptoms and sleep parameters. However, possibly due to low levels of comorbid psychiatric symptoms, functional disability and tiredness pre-treatment, it is impossible to estimate the potential clinical effectiveness of this intervention.

There are several limitations to the current study. First, as most participants were self-selected, the sample might not be representative of that in a clinical environment. Therefore, although the findings regarding feasibility of ICBT-I are promising and support the notion that internet-delivered programs for insomnia can be further developed in order to treat adolescents, it is unsure if the results can be generalized to a larger sample of participants and eventually implemented in health care. Second, the uncontrolled pilot design and with a limited sample size do not permit conclusions regarding causality and efficacy of the treatment. The results for changes in symptomatology following treatment should be considered preliminary and be interpreted with caution. Randomized clinical trials with rigorous protocols are necessary to validate the findings and the feasibility data suggest that the current clinical setting could be a fit for such large trial. Regarding treatment feasibility, substantial missing psychiatric data poses bias risk, highlighting data collection and participant engagement importance. Participants were reminded to complete assessments but uncompensated, potentially lowering completion rate. For a comprehensive trial, assessing data collection ease could curb missing data. As this study lacks follow-up data, initial completion insights for full-scale trial are absent. An important strength of the current study is the examination of ICBT-I in an age group for which a good evidence base of treatment is lacking. As the intervention is delivered by distance, the possible geographical reach of such treatment is not a limiting factor.

## Conclusion

The present study indicates that ICBT-I is feasible and accepted by an adolescent population. Preliminary results on efficacy indicates that insomnia symptoms and several sleep parameters can improve following the intervention, as well as depressive symptoms. These results are important, as untreated insomnia presents a risk factor for developing mental and physical health. To further investigate the potential use of ICBT-I in adolescents, large randomized controlled trials with different clinical populations should be performed, including the examination of comorbid symptoms and measures of daytime function. If the design and sample size of such studies would permit analyses of mediating and moderating factors, this would further add to the understanding of how insomnia in adolescents can be treated using ICBT-I.

## Supplemental Material

Supplemental Material - Internet-delivered cognitive behavioral therapy for adolescents with insomnia: Feasibility and preliminary efficacy Supplemental Material for Internet-delivered cognitive behavioral therapy for adolescents with insomnia: Feasibility and preliminary efficacy by Li Åslund, Susanna Jernelöv, Eva Serlachius, Sarah Vigerland, Rikard K Wicksell, Eva Henje and Mats Lekander in Clinical Child Psychology and Psychiatry
