# Supplementary material for: Internet-delivered cognitive behavioral therapy for adolescents with insomnia: Feasibility and preliminary efficacy
Source: Clin Child Psychol Psychiatry. 2023 Sep 12;29(3):1159–73. doi: 10.1177/13591045231202426 (PMC11188562; doi:10.1177/13591045231202426)
Supplement: Supplemental Material - Internet-delivered cognitive behavioral therapy for adolescents with insomnia: Feasibility and preliminary efficacy [file sj-pdf-1-ccp-10.1177_13591045231202426.pdf]

Supplementary Table 1. Caracteristics of the iSNOOZE-intervention.

Week 1	Week 2	Week 3	Week 4	Week 5	Week 6
Introduction to CBT-I.<br><br>Psychoeducation about sleep (e.g., sleep hygiene, function of sleep) and sleep disorders.<br><br>Describing the participant's problem, including functional analyses.<br><br>Goalsetting using SMART (specific, measurable, approved, realistic, timebound) goals.<br><br>Exercise: Functional analysis	Introduction of behavioral change.<br><br>Sleep restriction: using participant sleep-wake diary from week 1 to calculate bedtimes for sleep restriction.<br><br>Stimulus control.<br><br>Exercise: Goalsetting	Identifying behaviors of avoidance by functional analyses, including short/long term consequences.<br><br>Adjusting sleep restriction schedule.<br><br>Problem solving and introduction of alternative behaviors suitable for participant.<br><br>Exercise: Positive sleep behaviors	Identifying safety and avoidance behaviors.<br><br>Adjusting sleep restriction schedule.<br><br>Problem solving for overcoming obstacles (internal/external) related to treatment goals.<br><br>Strategies for dealing with sleepiness and worry related to this.<br><br>Introduction to a relaxation technique.<br><br>Exercise: Try a technique for relaxation	Dealing with obstacles in relation to individual treatment goals.<br><br>Adjusting sleep restriction schedule.<br><br>Functional analyses, identifying alternative behaviors and problem solving.<br><br>Exercise: Functional analyses and problem-solving	Summary of the treatment, repetition of important treatment components.<br><br>Strategies for preventing relapse: identifying successful components that the participants learnt, learning the difference between relapse and setback, identifying risky situations and behaviors to use, plan for continued work (next three months).<br><br>Exercise: Personal plan for relapse prevention
